# Mindfulness-based cognitive therapy (MBCT) versus the health-enhancement program (HEP) for adults with treatment-resistant depression: a randomized control trial study protocol

**DOI:** 10.1186/1472-6882-14-95

**Published:** 2014-03-11

**Authors:** Stuart J Eisendrath, Erin P Gillung, Kevin L Delucchi, Maggie Chartier, Daniel H Mathalon, Jude C Sullivan, Zindel V Segal, Mitchell D Feldman

**Affiliations:** 1 University of California, San Francisco Department of Psychiatry, 401 Parnassus Avenue, Box 0984-AIP, San Francisco, CA 94143, USA; 2 San Francisco VA Medical Center, 4150 Clement Street, San Francisco, CA 94121, USA; 3 University of Wisconsin, Madison Hospital & Clinics Sports Medicine Center, 621 Science Drive, Madison, WI 53711, USA; 4 Department of Psychiatry, University of Toronto, 250 College Street, Toronto, ON M5T1R8, USA; 5 University of California, San Francisco, Division of General Internal Medicine, 1545 Divisadero Street, Suite 315, San Francisco, CA 94143-0320, USA

**Keywords:** Depression, Treatment-resistant, Mindfulness, Meditation, MBCT

## Abstract

**Background:**

Major depressive disorder (MDD) is the leading cause of disability in the developed world, yet broadly effective treatments remain elusive. Up to 40% of patients with depression are unresponsive to at least two trials of antidepressant medication and thus have “treatment-resistant depression” (TRD). There is an urgent need for cost-effective, non-pharmacologic, evidence-based treatments for TRD. Mindfulness-Based Cognitive Therapy (MBCT) is an effective treatment for relapse prevention and residual depression in major depression, but has not been previously studied in patients with TRD in a large randomized trial.

**Methods/Design:**

The purpose of this study was to evaluate whether MBCT is an effective augmentation of antidepressants for adults with MDD who failed to respond to standard pharmacotherapy. MBCT was compared to an active control condition, the Health-Enhancement Program (HEP), which incorporates physical activity, functional movement, music therapy and nutritional advice. HEP was designed as a comparator condition for mindfulness-based interventions to control for non-specific effects. Originally investigated in a non-clinical sample to promote stress reduction, HEP was adapted for a depressed population for this study. Individuals age 18 and older with moderate to severe TRD, who failed to respond to at least two trials of antidepressants in the current episode, were recruited to participate. All participants were taking antidepressants (Treatment as usual; TAU) at the time of enrollment. After signing an informed consent, participants were randomly assigned to either MBCT or HEP condition. Participants were followed for 1 year and assessed at weeks 1–7, 8, 24, 36, and 52. Change in depression severity, rate of treatment response and remission after 8 weeks were the primary outcomes measured by the clinician-rated Hamilton Depression Severity Rating (HAM-D) 17-item scale. The participant-rated Quick Inventory of Depression Symptomology (QIDS-SR) 16-item scale was the secondary outcome measure of depression severity, response, and remission.

**Discussion:**

Treatment-resistant depression entails significant morbidity and has few effective treatments. We studied the effect of augmenting antidepressant medication with MBCT, compared with a HEP control, for patients with TRD. Analyses will focus on clinician and patient assessment of depression, participants’ clinical global impression change, employment and social functioning scores and quality of life and satisfaction ratings.

**Trial registration:**

ClincalTrials.gov identifier: NCT01021254

## Background

Major depressive disorder (MDD) is the number one cause of disability in developed countries and is projected to become the number two cause of disability worldwide by 2020 [1,2], yet broadly effective treatments remain elusive. The World Health Organization (WHO) Primary Care Study found that 60% of patients treated with antidepressants still met criteria for depression after one year of treatment [1]. Treatment-resistant depression (TRD), the term used to describe cases of MDD that do not remit with adequate courses of at least two antidepressant trials [3,4], is unfortunately quite common [3,5-8]. The seminal Sequenced Treatment Alternative To Relieve Depression (STAR*D) study found that only 30% of individuals with MDD will remit with one full trial of antidepressant medication, and the remission rates for successive trials are even lower [3,8]. TRD is associated with greater disability, mortality, morbidity, somatic symptoms, risk of relapse and societal cost than those who suffer from non-resistant forms of depression [4,9,10]. Hence, there is an urgent need for innovative and effective treatments.

Mindfulness-Based Cognitive Therapy (MBCT) is a group-based, 8-week, mind-body intervention that integrates mindfulness meditation with concepts of cognitive behavioral therapy (CBT) and was specifically developed as a relapse prevention intervention for MDD [11]. MBCT’s approach is distinctive, as it focuses on the cultivation of effective methods to relate to depressive thoughts and feelings, rather than challenging or changing specific cognitions as taught in CBT. MBCT teaches patients to relate to their unpleasant thoughts, feelings, and bodily sensations as temporary passing events in the mind, rather than identifying with them or treating them as accurate reflections of reality.

Several studies have investigated MBCT as an augmentative treatment for acute depression. As an augmentor to medications, results indicate that MBCT has similar efficacy to CBT [12,13]., MBCT augmentation was more efficacious than antidepressant management alone in reducing the residual symptoms of chronic depression [14,15] and improved outcomes in dysthymia compared to imipramine monotreatment [16]. Open label trials of MBCT augmentation of antidepressants for TRD showed effect sizes of 1.0 (Cohen’s D) in studies by both Kenny and Williams [12] and Eisendrath et al., [13], making the investigation of MBCT as a potentially effective augmentative treatment for TRD worth further examination in a full randomized controlled trial (RCT).

### Research objectives

The primary objective of this trial was to evaluate whether Mindfulness-Based Cognitive Therapy is an efficacious treatment for reducing depressive symptoms in adults with TRD. Individuals with TRD may be excellent candidates for MBCT [12,17], yet previous RCTs evaluating MBCT for depression failed to include an active comparator condition, limiting the results by not controlling for the non-specific factors (e.g., group support, homework, and facilitator attention) potentially associated with MBCT efficacy. With this limitation in mind, an active control condition, originally developed to be a structurally equivalent, comparison condition for studies Mindfulness-Based Stress Reduction MBSR) – the Health-Enhancement Program (HEP) was selected for this trial.

The study’s secondary objectives were to assess whether MBCT would be more effective than HEP in reducing symptoms of functional disability and improving overall quality of life and satisfaction and to examine potential mediators of MBCT treatment response. Finally, the study aimed to evaluate whether MBCT’s potential treatment effects related to depression severity, functional status, and mindfulness would be sustained during 1-year follow-up phase.

## Methods/Design

This study is a single-site, randomized, controlled clinical trial of MBCT augmentation of Treatment-as-Usual (MBCT + TAU) and HEP augmentation of TAU (HEP + TAU). TAU consisted of standard medication management for depression. In a single-blind fashion, participants were aware of treatment allocation but were blind to study hypothesis. Research assistants (RA’s) responsible for assessment of depression severity, were blind to individual randomization assignments. See Table 1 for a list of inclusion and exclusion criteria.

**Table 1:** Components of Mindfulness-Based Cognitive Therapy (MBCT) and the Health-Enhancement Program (HEP)

**Factor**	**MBCT**	**HEP**
Manualized intervention	X	X
Mindfulness training	X	
Music therapy		X
Nutrition education		X
Group support	X	X
Therapeutic attention	X	X
Session time (135 minutes)	X	X
Session duration (8 weeks)	X	X
Physical activity	X (e.g. yoga)	X (e.g. exercise, stretching)
Functional movement		X
Time equivalent homework	X	X
Facilitator buy-in to method	X	X

*Note.* X indicates the presence of each component in the intervention.

### Description of interventions

The two treatments, MBCT and HEP consisted of 8 weekly classes, each lasting 2.25 hours. In addition to attending weekly classes, participants in both groups were encouraged to complete 45 minutes of homework six days per week for the duration of the 8-week treatments. Both interventions were conducted in a group format with 6–12 participants per group and were led by two facilitators extensively trained in their respective techniques.

MBCT is a manualized treatment that combines training in mindfulness meditation with elements of cognitive therapy. For the purpose of this study, MBCT was adapted from the original manual developed by Segal et al. [11] with modifications for TRD, the details of which are published elsewhere [13]. These modifications are believed to promote enhanced emotional regulation specific to TRD through increasing an individual’s nonjudgmental awareness of their present moment experience [13]. MBCT teaches skills that encourage patients to disengage from habitual (“automatic”) dysfunctional cognitive routines, in particular depression-related ruminative thought patterns, as a way to reduce current symptoms, future risk of relapse and recurrence [16]. Through guided meditation practices and activities, participants are taught to develop more present-focused, non-judgmental awareness of their thoughts and feelings instead of focusing on the content of them [11,14,15,18]. It is theorized that the same cognitive processes known to make individuals prone to depression relapse are also active in perpetuating depression once it is established [12] and are a primary driver of TRD.

HEP was developed as a collaborative effort between the Waisman Laboratory for Brain Imaging and Behavior at the University of Wisconsin – Madison and the University of Wisconsin Hospital and Clinics Sports Medicine Center in consultation with the National Center for Complementary and Alternative Medicine (NCCAM). HEP was designed to provide health benefits to participants while omitting any components of mindfulness and was developed specifically as a control condition for studies of MBSR [19,20]. Originally used as a control condition for stress reduction in non-clinical samples, we adapted HEP to be an active comparison condition for individuals with TRD. HEP provides participants with intensive training in aerobic exercise (i.e. walking), functional movement, music therapy, and nutritional advice. Classes encourage participants to engage in experiential, didactic exercises which include exercise, functional movement, food journaling, song-writing, guided imagery and drawing. Participants are provided with psycho-educational lectures on topics related to aerobic exercise, novel functional movement patterns, nutrition, establishing a balanced diet, and how these health elements can impact mood.

Similar to the MBCT program, HEP participants were encouraged to individualize the program to meet their unique needs and symptoms. They were asked to attend all weekly sessions and engage in approximately 45 minutes of homework assignments on the remaining 6 days of the week. A portion of each class was devoted to participants describing their experience of the group and the “barriers” to treatment that arose in the completion of the formal and informal (i.e. homework) practices assigned by facilitators, which mirrors the same model used in MBCT. After the completion of the program, participants were provided with community resources to continue their practices, just as referral resources were provided during the final MBCT session.

In summary, HEP provides a credible comparator condition for MBCT by controlling for features of MBCT such as class participation, overall time invested, attention, group process, social interaction and support, homework, and experiential participation through in-class exercises (Table 1). A comparison of the course content by session for each intervention is outlined in Table 2. Study participants were not engaged in individual or group psychotherapy during the study treatment time period.

**Table 2:** Mindfulness-Based Cognitive Therapy (MBCT) and the Health Enhancement Program (HEP) Content by Session

**Session**	**MBCT**	**HEP**
1	Automatic Pilot: focuses on becoming aware in the present moment.	Problems in Improving Health: focuses on physical fitness, functional movement exercises, and music and imagery.
2	Dealing with Barriers: Participants discuss problems in focusing attention and are encouraged to adopt an attitude of “letting go” of self-criticism and judgment.	Physical Activity to Enhance Well-being: Physical activities focus on posture and alignment as ways to optimize function. Supportive music imagery exercises are used to improve sense of well-being.
3	Mindfulness of the Breath: Participants complete a sitting meditation in which they shift from focusing on body sensations to focus on their breath in a sitting posture. Mindful movement techniques are begun.	Understanding Dietary Guidelines: Participants engage in a lecture and discussion series about basic nutrition principals. Diet journaling is reviewed and demonstrated as a way to better understand healthy food choices.
4	Staying Present: Concepts of attachment/avoidance to pleasant/unpleasant sensations, moods, and thoughts are discussed. The “three-minute breathing space” is taught as a simple intervention for use throughout the day.	Fine-tuning Dietary Choices: Participants learn the use of the USDA* My Pyramid Food Tracker Program and behavioral changes are discussed as a way to meet dietary goals.
5	Allowing/Letting Be: Focus is on allowing experiences “to be” without judging or trying to change them.	Adapting to an Unpredictable Environment- Part I: Activities focus on functional movement and alignment. Supportive Music Imagery focuses on responding to the environment in creative ways.
6	Thoughts Are Not Facts: Focus is on viewing thoughts as mental events and not concrete facts.	Adapting to an Unpredictable Environment – Part II: Activities such as walking, jogging and stretching are discussed as participants are encouraged to respond to the environment in creative ways to enhance well-being.
7	How Can I Best Take Care of Myself: Participants learn their unique warning signs of depression relapse or worsening.	Responding to the Environment in Creative Ways: Participants learn the kinetic chain of functional movement and build group cohesion through group song writing exercises.
8	Using What Has Been Learned to Deal with Future Moods: Participants discuss how they will keep the momentum of their practice going. Therapists distribute information about settings where group mindfulness practice can be continued.	Support for Continuing Practice: Participants review the course and plan for maintaining gains including all components of HEP. Participants review their goals with the dietician.+

*USDA = United States Department of Agriculture.

+ The original HEP protocol was amended to include Nutrition in session 8.

TAU augmentation for both treatment groups was identical and consisted of medication management and supportive counseling delivered by third or fourth year psychiatric residents, attending psychiatrists or primary care physicians. Supportive therapeutic approaches aid in patients addressing problems without direct input from providers. Providers offer patients encouragement and guidance but do not engage in any therapeutic strategy other than active listening and offering support by focus on patients’ problems and concerns. Routine TAU visits are less than 30 minutes in duration and at intervals of one to three months. Participants were allowed to make medication changes during the acute and follow-up periods, with reporting of such changes on a regular basis. These changes will be incorporated into the analyses.

### Sample size

To estimate sample size, effect sizes were based on outcomes from previous studies and our pilot data [15,21-24]. In a review of 21 mindfulness-meditation interventions, Baer [23] found an average effect size of .90 for depressive symptom reduction. In examining the relapse rates of individuals receiving MBCT versus TAU, Teasdale et al. [14] found an effect size of .59 and Ma et al. [15] of .88 for participants with a history of 3 or more depressive episodes. Our pilot data indicated an average effect size of 1.13, which compared similarly to Kenny’s effect size of 1.03 in a TRD population [12]. Finucane et al., working with a more severely depressed population, found an effect size of 1.54 [25]. Kingston et al. found an effect size of 1.07 in reducing depression in psychiatric outpatients [26]. There were no significant differences in effect on depression in a comparison of HEP and MBSR [19,20]. Using the formula provided by Donner and Klar [27], we estimated sample sizes to detect response to the intervention (MBCT + TAU would be superior to HEP + TAU on depression outcomes). Results indicated sample sizes ranging up to approximately 50 per condition. A total sample of 124 participants were regarded as necessary to provide 80% power to detect a moderate effect size in the face with an estimated attrition of approximately 20%, similar to our rates of attrition in clinical MBCT groups.

### Recruitment

Study recruitment was conducted between September 2009 and September 2013 in outpatient psychiatry and general medicine clinics at the University of California, San Francisco (UCSF) Medical Center and the outpatient psychiatry clinic at Kaiser Permanente in San Francisco. Both recruitment sites were housed in large medical centers that serve an ethnically diverse population of privately and publically insured adults in the San Francisco Bay Area. To further contribute to the diversity of the patient sample, community locations were added to the recruitment plan at later stages in the study, as well as clinics at San Francisco General Hospital.

Recruitment methods were approved by the institutional review board (IRB) at the UCSF Human Research Protection Program (#10-00455). For patients served through general medicine clinics at UCSF, approval was obtained to introduce the study with a letter from the principal investigator (PI) to prospective research subjects who had been identified through a query of patient medical record databases to have an ICD-9 code of depression and/or a medical history of taking antidepressant medication. Patients receiving the letter were invited to opt out of the study by mail within two weeks of receiving the letter. Patients who chose not to opt out were contacted by telephone by a RA to assess interest and eligibility to participate in the study. Potential candidates from other recruitment sites were referred by various methods, including research flyers, referrals from treating physicians, online announcements and print advertisements posted in public spaces and inside of buses and other public transportation vehicles in the Bay Area.

### Eligibility criteria (inclusion and exclusion criteria)

Participants age 18 or older were required to meet DSM-IV criteria for major depressive disorder and have a total score of 14 or higher on the clinician-rated, 17-item version of the Hamilton Depression Severity Rating Scale (HAM-D) [28] to be eligible for the study. To meet criteria for TRD, participants were required to be taking antidepressant medications with evidence of at least two adequate antidepressant trials prescribed during the current depressive episode. Participants were excluded (Table 3) from the study for the following reasons: they a) met DSM-IV criteria for bipolar disorder, obsessive compulsive disorder, or post-traumatic stress disorder (other comorbid Axis I conditions were noted but not cause for exclusion); b) had a history of schizophrenia or other psychotic symptoms; c) were imminently suicidal, a danger to others or were currently exhibiting self-injurious behavior; d) met DSM-IV criteria for alcohol or substance abuse or dependence within 3 months of study entry; e) were currently practicing meditation more than once per week or yoga more than two times per week; f) had cognitive impairment as evidenced by a score of < 25 on the MMSE; g) were in concurrent individual or group psychotherapy and were not willing to discontinue treatment for the 8-week duration of study treatment; h) had significant or unstable medical illness that would limit participation in HEP; or i) did not have adequate English language comprehension.

**Table 3:** Eligibility criteria for the PATH-D study

**Inclusion criteria**	**Exclusion criteria**
- Age 18 or older	- Clinically significant suicide risk
- Current major depression (DSM-IV-TR criteria)	- History of schizophrenia or psychotic symptoms
- HAM-D total score ≥ 14	- Bipolar disorder
- Total ATHF Intensity score ≥5 for 2 or more antidepressant medications	- Obsessive compulsive disorder
- Evidence of two failed adequate antidepressant trials in the current depressive episode	- Alcohol or substance abuse or dependence within the past 3 months
- Current use of antidepressants	- Insufficient ability to understand or read English
	- MMSE total score < 25
	- Severe medical illness determined by the CIRS
	- History of meditation practice or currently meditation more than 1 time per week
	- Currently practicing yoga more than 2 times per week

*Note.* HAM-D = Hamilton Depression Rating Scale; ATHF = Antidepressant Treatment History Form; MMSE = Mini Mental Status Exam; CIRS = Cumulative Illness Rating Scale.

*Axis I anxiety disorders are not excluded as they are highly co-morbid with depression. This study is designed to approximate a real world, clinical setting, and anxiety disorders were not excluded in the original MBCT studies nor in the STAR*D trials.

### Assessment of eligibility and informed consent

Interested individuals underwent initial screening for eligibility using a brief, structured telephone interview. Those with depressive symptoms being treated with antidepressants medications and not meeting exclusion criteria in Table 3 were invited for an in-person screening evaluation to determine eligibility and review informed consent. Psychological eligibility criteria such as depression severity and psychiatric history were assessed by a licensed mental health professional on the research team who had undergone intensive standardized training provided by one of the authors. Prior to the first assessment session, RAs explained the trial to participants by reviewing the study procedures, treatment interventions and required time commitment, and participants were given an opportunity to ask questions before signing informed consent. Participants were reminded that study participation was voluntary and that they had the right to withdraw from the research at any time without disruption to their usual care.

During the screening assessment, consenting participants provided information about their socio-demographic status. Assessment of depression severity and psychiatric history was conducted through a semi-structured clinical interview using the Structured Clinical Interview for DSM-IV (SCID) [29], the HAM-D [28] and the Clinical Global Impression Severity (CGI-S) [30] scale. The SCID personality inventory for Axis II disorders (SCID-II) was used to assess the presence of personality disorders, and the Mini Mental Status Exam (MMSE) [31] was used to assess cognitive function and to rule out cognitive or developmental delays that might interfere with treatment. Treatment history and adequacy of antidepressant medication treatment was evaluated using the Antidepressant Treatment History Form (ATHF) [32]. Confirmation of at least 2 adequate trials of efficacy with a minimum total intensity score of 5 points or greater was required for study entry. In cases where treatment history or medication compliance was in question, RAs received informed consent from participants to review medical records and/or contact prescribing physicians to confirm adequacy of treatment. Finally, participants were asked to complete the self-report Cumulative Illness Rating Scale (CIRS) [33,34] to assess health status. Scores of 4 or more on any one item required further evaluation from the research team to ensure participants could perform the required physical activities in HEP. Table 4 outlines all measurements and a complete list of time points of administration.

**Table 4:** Eligibility and outcome measures

**Category/**	**Week of measurement point**												Method of measurement*
**Measure**	**# Item**	**Screen**	**0**	**1**	**2**	**3**	**4**	**5-7**	**8**	**24**	**36**	**52**	
**Eligibility and demographic measures**													CI
Contact Info\Demographic data	-	X											CI
Mini mental status exam	11	X											CI
Structured clinical interview for diagnosis of axis I/II	-	X											CI
Antidepressant treatment history form	-	X											CI
Cumulative illness rating scale		X											CI
**Depression outcomes**													
Hamilton depression rating scale	17	X	X				X		X	X	X	X	CI
Quick inventory of depression symptomatology	16		X	X	X	X	X	X	X	X	X	X	PQO
Clinical global impressions scale	2		X				X		X	X	X	X	CI
SCID mood module for major depression	-								X	X	X	X	CI
Longitudinal interval follow-up evaluation	-								X	X	X	X	CI
**Functional status outcomes**													
Health status-short form 12	12		X				X		X	X	X	X	PQO
Work and social activity scale	5		X				X		X	X	X	X	PQO
Quality of life enjoyment satisfaction questionnaire	15		X				X		X	X	X	X	PQO
**Measures of mediation/moderation**													
Therapeutic rationale scale	4				X				X				PQO
State trait anxiety inventory	40		X				X		X	X	X	X	PQO
Perceived stress scale	14		X				X		X	X	X	X	PQO
Childhood trauma questionnaire		X											PQO
Ruminative response scale	22		X				X		X	X	X	X	PQO
Acceptance and avoidance questionnaire	10		X				X		X	X	X	X	PQO
Five facet mindfulness questionnaire	30		X				X		X	X	X	X	PQO
Experiences questionnaire	20		X				X		X	X	X	X	PQO
Self-compassion scale	26		X				X		X	X	X	X	PQO
Medication/Psychotherapy change form	-		X	X	X	X	X	X	X	X	X	X	CI
Homework summary log	-		X		X	X	X	X	X	X	X	X	PQO

*CI = Clinical interview; PQO = Patient questionnaire online.

Data obtained during the intake interview was presented in a weekly research conference where the principal investigator reviewed eligibility criteria on a case-by-case basis to make determinations about participants’ eligibility for enrollment.

### Randomization and baseline assessment

**Figure 1:**
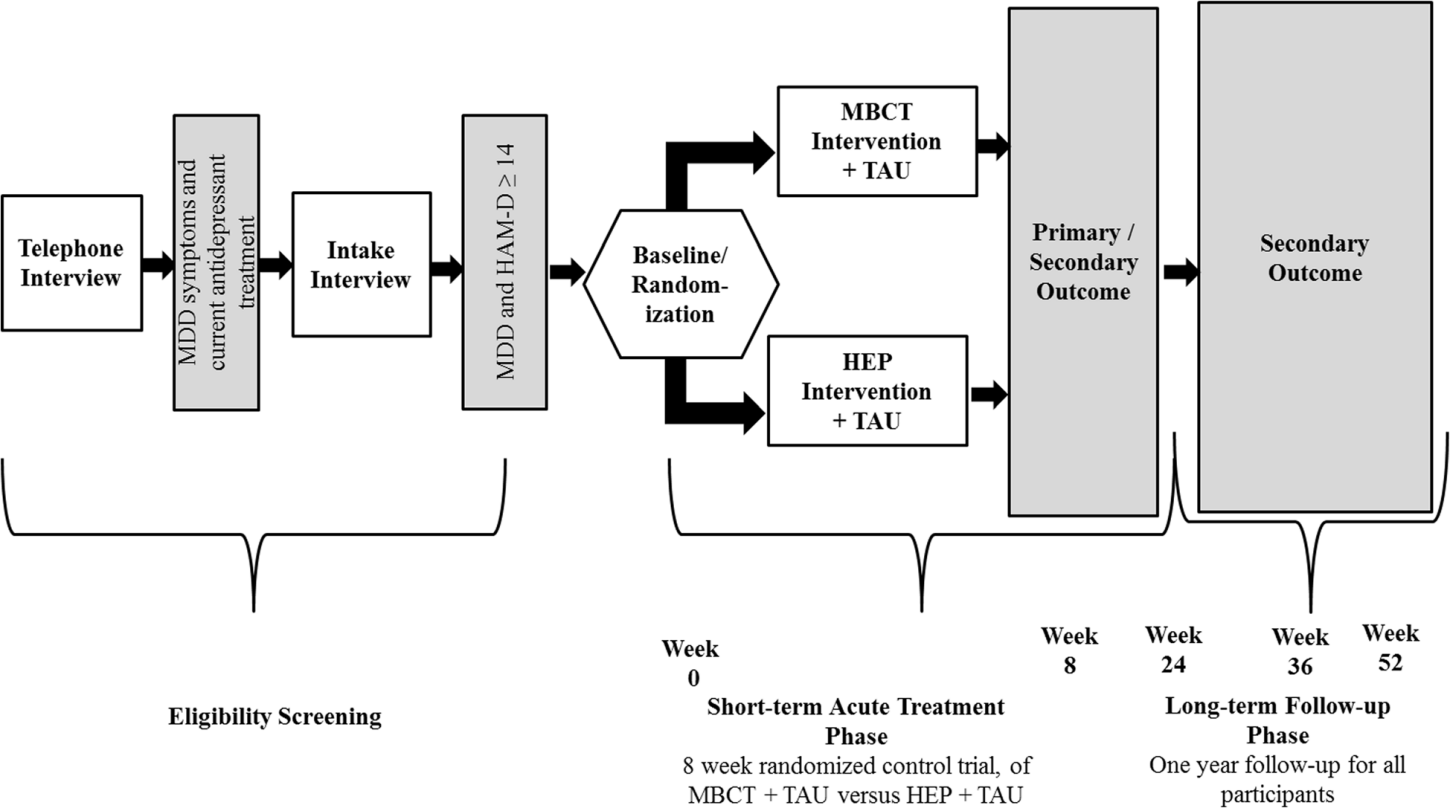
Study design of randomized control trial of Mindfulness-Based Cognitive Therapy (MBCT) versus the Health-Enhancement Program (HEP) in adults with treatment-resistant depression.

The unit of randomization was the patient, and randomization tables were stratified by gender to ensure balanced assignment. Patients were randomized during the baseline assessment, which occurred within two weeks of the commencement of treatment (see Figure 1. Study Design and Participant Flow). Baseline assessments served two purposes: 1) to ensure that patients continued to meet eligibility criteria for MDD with scores ≥ 14 on the HAM-D and 2) to serve as the primary data collection point (see Outcome Measures section). Patients who reported improved depressive symptoms during the baseline interview with HAMD total scores < 14, were not randomized to treatment and told that they could be reassessed for a future intervention cohort should their depression symptoms relapse. Participants who continued to be eligible on measures of depressive severity were then randomly assigned to either MBCT + TAU or HEP + TAU.

### Outcome measures

Outcomes were assessed at baseline, weeks 1–7, at the end of treatment (week 8) and at weeks 24, 36 and 52 during the long-term follow-up phase. Data was obtained from patient questionnaires completed online and through clinical RA ratings, which were confirmed with chart review of medical records when necessitated.

### Primary and secondary outcome measures

The primary outcome measurement was improvement of depression severity after 8 weeks, as measured through change in HAM-D total scores from pre- to post-treatment, treatment response (scores ≥ 50% decrease from baseline) and remission (post-treatment scores ≤ 7). Length of treatment efficacy, a secondary outcome, was assessed up to 1 year after baseline using HAM-D scores, response and remission rates by measuring rate of depression relapse using the semi-structured Longitudinal Interval Follow-up Evaluation (LIFE) [35]. The participant-rated 16-item Quick Inventory of Depressive Symptomatology (QIDS) [36] was used as a secondary measurement to assess change in depressive severity, response (scores ≥ 50% decrease from baseline) and remission (scores ≤ 5) during the same treatment and follow-up time period.

Functional status and quality of life were additional secondary outcomes measured using the SF-12 Health Survey [37,38], the Work and Social Adjustment Scale (WSAS) [39], [40] and the Quality of Life, Enjoyment, and Satisfaction Questionnaire (Q-LES-SQ) [41].

#### Mediators, moderators and potential covariates

A number of other psychometric variables believed to be associated with mindfulness were investigated and measured at baseline, during mid-point (week 4), after the end of treatment (week 8) and the long-term follow-up weeks 24, 36, and 52. The following potential mediators related to MBCT were measured: mindfulness with the Five Facet Mindfulness Questionnaire [42]; experiential avoidance with the Acceptance and Action Questionnaire (AAQ) [43]; rumination with the Ruminative Response Scale (RRS) [44,45]; and self-compassion with the Self-Compassion Scale (SCS) [46].

Psychiatric factors that may impact depression severity and resistance such as early age of depression onset, number of recurrences, poor treatment outcomes (e.g. incomplete remission) and co-morbid psychiatric illness (e.g. anxiety disorders [47] or personality disorders [48]), will be included in the analysis as covariates [49]. Also included will be other factors known to be moderators of depression treatment outcomes. These include demographic factors such as minority status, ethnicity and lower socio-economic status [50] and stress, both current [51,52] and a childhood history of stress [53] (trauma, abuse, neglect) which were collected during the baseline assessment.

Medication changes in the TAU arm are the same ones utilized in treating persisting or worsening depression in the STAR*D studies [3,8]. Changes were tracked during the 8-week treatment and over the 52 week follow-up through patient report and chart review. Alternate forms of treatment (i.e. individual psychotherapy, group psychotherapy, electroconvulsive therapy, etc.) for depression was tracked after the treatment-phase of the study through patient report and interview. These treatments will be analyzed as covariates as well as secondary outcomes, similar to the aforementioned medication changes.

Treatment expectancy to assess potential moderating effects of positive expectancy was measured through the Treatment Rationale Scale [54-56], which participants completed after the first and final sessions (weeks 2 and 8). Changes in psychotropic medications including antidepressants, benzodiazepines and mood stabilizers were tracked at every assessment time point using a medication evaluation form. Lastly, participant engagement in treatment was measured through attendance and total time spent completing homework and related activities outside of class. A complete list of the outcome measures, measurement points and assessment methods for the study are summarized in Table 4.

#### Treatment adherence and fidelity assessment

Adherence to treatment was defined by measuring the received therapy dose through number of sessions attended. In the current trial, a completer of treatment was defined as participation in at least five out of eight sessions.

Group leaders digitally recorded all treatment sessions, and recordings were sent to independent evaluators not affiliated with the university or part of the research team. MBCT adherence and competency was provided by an expert in MBCT who reviewed 3 randomly selected sessions from each treatment cohort. For the control condition, external monitoring of treatment HEP adherence and facilitator competency was completed by a HEP expert who also reviewed 3 randomly selected sessions from each treatment cohort. Internal treatment adherence and competency for both interventions was assessed by the principal investigator who reviewed the same 3 randomly selected sessions. The MBCT Adherence Scale [57], an instrument used in the original studies of MBCT, was used to assess treatment fidelity. Inter-rater reliability ranges from 0.59 for the Cognitive Therapy subscale and .97 for the Mindfulness subscale, and 0.82 for global ratings. Competence in delivering MBCT is rated with a newly developed measure, MBCT Competence Scale [13]. This 10-item instrument uses a scale from 0–5 to assess each facilitator’s skill in delivering the treatment. Treatment Fidelity in HEP was assessed using the HEP Adherence Scale. The HEP Adherence Scale is a newly developed scale and is modeled after the MBCT Adherence Scale. It is a 17-item instrument where items are score from 1 to 3. Competence in delivering HEP was rated with a newly developed instrument, the HEP Competency Scale. This is a 10-point scale for rating group facilitator skill and expertise in delivering the intervention. Because the HEP Adherence Scale, HEP Competency Scale, and MBCT Competency Scale have not been previously validated, they will serve as templates for assessment in this study and pilot data for future research. Based on reviews and results of adherence and competency measures, feedback was provided to group leaders during weekly supervision meetings.

### Data safety and monitoring plan

In consultation with NCCAM, an independent Data Safety Monitoring Board (DSMB) was established to oversee all study activities to ensure the safety of participants, the validity of our findings and the need for further data collection. Three categories of patient safety concerns were highlighted for this study: 1) increased depression severity; 2) risk to safety through self-harm and increased suicidal ideation and; 3) study withdrawals that were potentially or definitely related to specific factors in each treatment intervention. Regarding suicidal ideation among enrolled participants, when patients responded positively to the suicidality questions on the HAM-D and QIDS or experienced a 1 or greater point increase from their baseline scores, RAs initiated the following actions they: 1) they notified the PI; 2) provided patients with referrals to crisis hotlines; 3) discussed with patients whether the patient had notified their prescribing physician or mental health provider about their symptoms and; 4) in some cases, where the PI deemed that more clinical contact was warranted, they corresponded via telephone and/or sent confidential letters and email correspondences to providers notifying them of their clinical impressions.

### Planned analytic approach

The primary outcome measure of the HAM-D will be compared between participants randomized to the MBCT + TAU and the HEP + TAU groups using a mixed-effects statistical model [58,59]. Mean levels of severity, and response and remission rates at week 8 and bivariate measures will be compared between treatment conditions using logistic regression models with generalized estimating equations to correct for interdependence resulting from group membership. The secondary outcome measures of the QIDS-SR and CGI will be compared using the same methodology but replacing the HAM-D with the QIDS-SR and CGI severity measures. We will also assess relapse occurrence in both arms using the LIFE interview at weeks 24, 36 and 52.

The efficacy of MBCT + TAU versus HEP + TAU on functional status as a secondary outcome will be assessed using similar models to the methods used to test the reduction in HAM-D_17_ scores. For each of the four scales, scores at week 8 will be compared between randomized conditions using a mixed-effects statistical model, which will include effects for randomized condition, therapy group and baseline disability score. The test of the estimated parameter for treatment condition will be a direct test of this hypothesis. Mediating effects of the relationship of treatment assignment with outcome will be tested using a series of linear regression models. Putative mediators, enhanced mindfulness (FFMQ), enhanced self-compassion (SCS), diminished rumination (RRS) and decreased experiential avoidance (AAQ) will be assessed at weeks 4, 8, 24, 36, and 52. We will also assess persistence of treatment changes in depression (HAM-D, QIDS-SR, CGI), functional status (SF-12, WSAS, Q-LES-Q-SF), mindfulness (FFMQ), rumination (RRS), self-compassion (SCS) and avoidance (AAQ) at weeks 24, 36 and 52 by analyses parallel to those outlined for testing Hypotheses 1 and 2 using data from the weeks 24, 36, and Week 52. Mixed-effects and logistic regression models using a generalized estimating equation (GEE) will allow direct testing of these effects. Moderating factors (e.g. age of depression onset, prior poor treatment outcomes, co-morbid psychiatric diagnoses and other socio-demographics) associated with TRD will also be included in the model.

Additional secondary analyses will test for effects of time. These models will also include indicator variables of any additional treatments (e.g. HEP participants’ crossing over to take MBCT outside of the research), medication changes after the end of acute treatment before the 24, 36 and 52-week follow-ups and also whether or not patients began new psychotherapeutic interventions after treatment or continued formal meditation practices.

Based on the extent and nature of missing data (outcomes, predictors, baseline, and items from scales), we will use one of several strategies for data analysis. We anticipate that the majority of missing data will result from participant attrition. In this case, we will know the randomization assignment and have data from some, but not all, of the planned assessments. Our primary approach to dealing with this monotone missing data pattern will be a pattern-mixture modeling approach [60]. We may also be missing some data on predictors, even in the absence of participant attrition. Our primary method for dealing with missing predictor values will be multiple imputation using a regression modeling approach [60]. This method is superior to simpler methods such as using group means for missing data. In order to limit attrition, we will provide an incentive in the form of an assessment payment schedule to increase motivation to complete all assessments.

## Discussion

Treatment-resistant depression is a condition associated with high rates of disability, chronicity, reoccurrence and failed response to antidepressant treatment [3,8]. Through decreased workforce productivity and disability, the economic burden caused by refractory depression is enormous [10,61]; hence the importance of finding effective treatment modalities for this population is clear and of vital public health interest. This investigation represents the first RCT to evaluate MBCT for TRD. Results from this study will address whether MBCT is superior in reducing depression severity after 8 week treatment and improving relapse and remission rates over time in comparison to a credible comparator condition, developed to control for non-specific treatment effects. If successful, this study will identify an augmentation treatment for a large, hard-to-treat population for whom few treatments have been effective.

After analysis, we will be able to assess whether the specific constructs of a mindfulness-based intervention, such as the putative mediators in MBCT (increased mindfulness, self-compassion and decreased rumination and experiential avoidance) are enhanced more after an 8-week treatment with MBCT compared with HEP, and whether they are associated with positive treatment outcomes. We will also be able to evaluate whether treatment gains between groups (i.e. reduced depression severity, increased functional status) are retained during a 1-year follow-up and whether differential effects might be mediated by the specific constructs of MBCT listed above. Finally, we will be able to assess whether participant expectancy, treatment adherence (i.e. number of sessions attended), and compliance (i.e. number of minutes of homework completed) predicted treatment outcomes.

### Limitations

The TAU intervention has some variance because we allowed medication changes to take place, which increases the possibility of wash-out effects of MBCT or falsely amplifying its effects. Moreover, antidepressants alone are also well documented to be associated with placebo effects, although these appear to be reduced in a TRD population [62]. Because our aim was to show that MBCT is an effective augmentation strategy and because we wanted trial results to be widely generalizable in light of routine clinical practice, we permitted some flexibility in medication changes. We anticipate that randomization and statistical analysis will control for this variance and allow us to compare the impact of medication changes in both arms.

The current investigation lacks a 3^rd^ arm or wait-list control condition, which limits the ability to determine whether factors unrelated to treatment, such as time or life events effects were associated with treatment effects. We will however, be able to compare our outcomes to similar TRD populations presented by Kenny and Williams [12] and Eisendrath et al., [13]. While we expect the association to be modest, we predict that treatment expectancies or patients’ initial beliefs about the success of either intervention could influence treatment outcomes. The current investigation lacks a measure of treatment preference, which could have played a predictive role in affecting clinical outcomes.

### Summary

In summary, the results of this trial will address whether MBCT, previously shown to be effective for relapse prevention, may be an effective augmentation therapy for patients with TRD in active depressive episodes. In addition, this is the first RCT to compare whether MBCT is more effective in reducing depression, rates of relapse and disability when augmented by medications (TAU) than an active comparator condition; thereby isolating the elements of MBCT that might be most salient. In particular, the study will highlight whether mindfulness or other features of the interventions play important roles in the outcomes. In addition, the study will shed light on which MBCT factors play mediating roles in producing its effects. National self-report surveys have found complementary and alternative techniques such as mindfulness, are being used by 41% to 54% [63] of individuals with depression, indicating that there is substantial public demand for mindfulness-based treatment interventions. Consequently we believe the results of this study will have significant applications for depression care by informing providers about targeted, population-based non-pharmacological interventions strategies that may also be appealing to TRD patients.

## Abbreviations

TRD: Treatment-resistant depression; MBCT: Mindfulness-based cognitive therapy; HEP: Health-Enhancement Program; TAU: Treatment as usual; CBT: Cognitive behavioral therapy; RA: Research assistant; MBSR: Mindfulness-Based Stress Reduction; IRB: Institutional review board; PI: Principal investigator; DSMB: Data safety monitoring board; GEE: Generalized estimating equation.

## Competing interests

The authors declare that they have no competing interests.

## Authors’ contributions

SJE developed the original study design and secured funding. SJE and EPG drafted the primary manuscript. KLD contributed the sample size calculation and data analysis plan for reporting of study results. SJE, EPG, MC, JCS, DHM, KLD, ZVS and MF participated in editing this paper. All authors substantially revised the article, read and approved the final manuscript.

## Pre-publication history

The pre-publication history for this paper can be accessed here:

http://www.biomedcentral.com/1472-6882/14/95/prepub
